# Analysis of Gene Regulatory Networks in the Mammalian Circadian Rhythm

**DOI:** 10.1371/journal.pcbi.1000193

**Published:** 2008-10-10

**Authors:** Jun Yan, Haifang Wang, Yuting Liu, Chunxuan Shao

**Affiliations:** CAS-MPG Partner Institute for Computational Biology, Shanghai Institutes of Biological Sciences, Shanghai, China; Pennington Biomedical Research Center, United States of America

## Abstract

Circadian rhythm is fundamental in regulating a wide range of cellular, metabolic, physiological, and behavioral activities in mammals. Although a small number of key circadian genes have been identified through extensive molecular and genetic studies in the past, the existence of other key circadian genes and how they drive the genomewide circadian oscillation of gene expression in different tissues still remains unknown. Here we try to address these questions by integrating all available circadian microarray data in mammals. We identified 41 common circadian genes that showed circadian oscillation in a wide range of mouse tissues with a remarkable consistency of circadian phases across tissues. Comparisons across mouse, rat, rhesus macaque, and human showed that the circadian phases of known key circadian genes were delayed for 4–5 hours in rat compared to mouse and 8–12 hours in macaque and human compared to mouse. A systematic gene regulatory network for the mouse circadian rhythm was constructed after incorporating promoter analysis and transcription factor knockout or mutant microarray data. We observed the significant association of *cis-*regulatory elements: EBOX, DBOX, RRE, and HSE with the different phases of circadian oscillating genes. The analysis of the network structure revealed the paths through which light, food, and heat can entrain the circadian clock and identified that NR3C1 and FKBP/HSP90 complexes are central to the control of circadian genes through diverse environmental signals. Our study improves our understanding of the structure, design principle, and evolution of gene regulatory networks involved in the mammalian circadian rhythm.

## Author Summary

Circadian rhythm is universally present from unicellular organisms to complex organisms and plays an important role in physiological processes such as the sleep–wake cycle in mammals. The mammalian circadian rhythm presents an excellent system for studying gene regulatory networks as a large number of genes are undergoing circadian oscillation in their expression levels. By integrating all available microarray experiments on circadian rhythm in different tissues and species in mammals, we identified a set of common circadian genes lying in the center of the circadian clock. Significant differences in the circadian oscillation of gene expression among mouse, rat, macaque, and human have been observed that underlie their physiological and behavioral differences. We constructed a gene regulatory network for the mouse circadian rhythm using knockout or mutant microarray data that have previously received little attention. Further analysis revealed not only additional feedback loops in the network contributing to the robustness of the circadian clock but also how environmental factors such as light, food, and heat can entrain the circadian rhythm. Our study provides the first gene regulatory network of the mammalian circadian rhythm at the system level. It is also the first attempt to compare gene regulatory networks of circadian rhythm in different mammalian species.

## Introduction

Circadian rhythm is a daily time-keeping mechanism fundamental to a wide range of species. The basic molecular mechanism of circadian rhythm has been studied extensively. It has been shown that the negative transcriptional–translational feedback loops formed by a set of key circadian genes are responsible for giving rise to the circadian physiology. In mammals, the master clock resides in the suprachiasmatic nucleus (SCN) and the SCN orchestrates the circadian clocks in peripheral tissues by directing the secretion of hormones such as glucocorticoids. Through many years of molecular and genetic studies, at least 19 key circadian genes—*Per* family (*Per1/Per2/Per3*), *Cry* family (*Cry1/Cry2*), *Bmal1* (*Arntl*), *Clock*, *Npas2*, *Dec1/Dec2* (*Bhlhb2/Bhlhb3*), *Rev-erbα/β* (*Nr1d1/Nr1d2*), *Rora/Rorb/Rorc*, *Dbp/Tef/Hlf*, and *E4bp4* (*Nfil3*)—have been identified in mammals [1]. As is now commonly accepted, Arntl and Clock proteins form a complex that positively regulates the transcription of *Per* and *Cry* family genes through activating the *cis-*regulatory element E-box in their promoters. Per and Cry family proteins form a complex that inhibits Arntl/Clock transcriptional activity, thus completing the negative feedback loop. Other key circadian genes such as *Dbp* and *Nfil3* controlling the D-box element and *Rora*/*Rorb*/*Rorc* and *Nr1d1*/*Nr1d2* controlling the RRE (Rev-erb/Ror element) have also been shown to be important to the mammalian circadian rhythm.

Since 2002, there have been a series of microarray experiments aimed at identifying circadian oscillating genes at the genome-wide level in various tissues of mammalian species, including mouse, rat, rhesus macaque, and human (Table S1). These experiments usually identified hundreds of circadian oscillating genes, suggesting that the circadian rhythm drives a genomewide circadian oscillation of gene expression. However, microarray data are intrinsically noisy, and further, these microarray experiments differed in the animals that they used, experimental conditions, and sampling times, *etc*. Indeed, these microarray experiments have so far not been compared or integrated. In a few cases where two tissues were studied in a single experiment, the overlap of circadian oscillating genes between tissues was very limited [2],[3]. Assuming that a set of common circadian genes exists in most tissues and cell types, integration of different circadian microarray datasets in multiple tissues could potentially identify such a common set of circadian genes [4]. Comparison of circadian oscillating genes and their oscillating patterns across different tissues can help us understand the tissue-specific functions of circadian rhythm. Comparison across different mammalian species can also shed light on the molecular mechanisms that lead to their different physiologies and behaviors.

Because many known key circadian genes such as *Arntl*/*Clock*, *Nr1d1*/*Nr1d2*, and *Dbp*/*Nfil3* are transcription factors, transcriptional regulation must have played an important role in the genome-wide circadian oscillation of gene expression. Ueda et al. constructed a small-scale gene regulatory network consisting of 16 genes and 3 *cis-*regulatory elements based on in vitro luciferase reporter assays [5]. However, the construction of a circadian gene regulatory network at the system level based on promoter analysis alone has been almost impossible due to the difficulties in transcription factor binding site prediction [6]. The existence of other *cis-*regulatory elements associated with circadian oscillation has remained elusive. On the other hand, there are a large body of microarray experiments from transcription factor knockout or mutant animals currently available at public databases. Incorporating the knockout or mutant microarray experiment results with the promoter sequence analysis can greatly facilitate the identification of functional transcription factor binding sites. In general, construction and analysis of gene regulatory networks involved in the mammalian circadian rhythm will improve our understanding on how key circadian genes are driving circadian-controlled genes, and will pave the way for more detailed quantitative modeling of the mammalian circadian rhythm.

## Results

### Identification of a Common Set of Circadian Genes in Mouse

**Figure 1:**
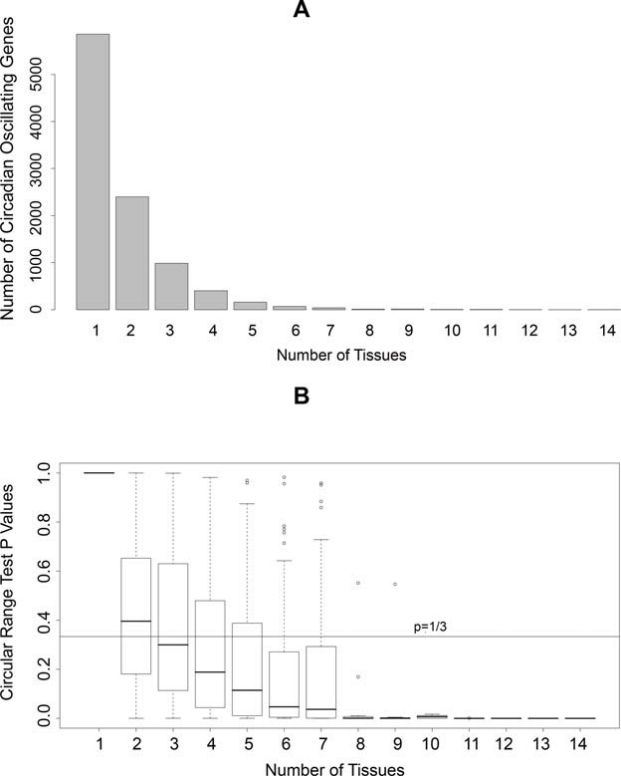
Tissue distribution of circadian oscillating genes. (A) Distribution of the number of circadian oscillating genes identified in different numbers of mouse tissues. (B) Distribution of *p*-values in circular range tests for circadian phases of circadian oscillating genes identified in different numbers of mouse tissues.

We searched for circadian oscillating genes in 21 circadian time series microarray data covering 14 tissues in mouse (Table S1) by fitting them to cosine functions with different phases, and extracted circadian phase information for circadian oscillating genes. We identified 9,995 known genes showing circadian oscillations in at least one tissue (Table S2). The number of genes showing circadian oscillation in multiple tissues decreases rapidly as the number of tissues increases, whereas the consistency of their circadian phases across tissues as measured in *p*-values of circular range tests improves rapidly (Figure 1). We identified 41 common circadian genes, defined as the genes showing circadian oscillation in at least 8 out of 14 tissues in mouse (Table 1). 13 out of 19 previously known key circadian genes were among the common circadian genes that we identified in this study. Other known key circadian genes: *Rorb*, *Cry2*, *Rora*, *Npas2*, and *Hlf* were found to be circadian oscillating in one, three, three, four, and five tissues, respectively. *Bhlhb3* was not found to be circadian oscillating in any tissue. 39 of these common circadian genes showed significant consistency (*p<*1/3 in circular range test) of their circadian phases across all tissues.

**Table 1:** Circadian phases of common circadian genes in 14 mouse tissues.

Gene Symbol	SCN	LIV	KID	AOR	SKM	HAT	ADG	BAT	WAT	BON	PFR	WB	ATR	VEN
1500005K14Rik	NA	NA	22.5	1.8	21.7	0.2	3.7	22.7	NA	22.0	2.5	3.8	NA	NA
Arntl	16.8	22.3	22.2	22.0	23.0	0.5	22.6	21.5	22.3	22.3	NA	0.6	22.5	22.7
Bhlhb2	5.2	12.8	12.7	NA	NA	7.7	12.8	NA	NA	8.3	14.7	15.8	6.5	6.8
Ccrn4l	8.7	12.8	13.0	9.3	18.0	NA	10.2	NA	NA	15.0	NA	NA	12.8	NA
Cdkn1a	NA	22.3	NA	18.8	4.0	NA	23.8	20.3	22.7	0.8	16.7	23.0	23.2	NA
Cirbp	9.0	3.5	NA	5.2	NA	NA	6.7	4.7	5.0	4.2	NA	8.8	NA	NA
Clock	NA	22.3	21.2	19.8	NA	1.2	0.3	NA	22.3	23.2	NA	1.7	0.7	0.7
Col4a1	NA	2.2	NA	2.2	2.2	2.7	NA	1.2	3.7	1.8	NA	NA	0.5	1.0
Cpt1a	NA	9.0	7.2	6.7	0.0	5.7	9.3	9.3	7.0	5.1	NA	NA	NA	NA
Cry1	10.7	19.6	17.2	18.5	18.5	NA	18.0	19.8	19.5	18.2	15.7	18.6	NA	NA
Dbp	5.5	9.1	NA	8.8	10.0	10.3	9.1	9.2	9.9	9.4	NA	11.4	8.3	8.7
Fbn1	NA	NA	NA	2.3	1.3	3.2	4.8	NA	1.1	3.8	NA	NA	0.8	1.0
Fkbp5	NA	14.2	14.7	14.5	3.8	NA	13.3	14.5	12.7	NA	11.2	13.8	15.7	NA
Gsta3	NA	19.1	NA	17.3	NA	15.8	18.3	20.7	18.7	20.8	NA	NA	15.3	14.0
H3f3b	1.5	3.1	5.5	NA	NA	NA	4.2	4.2	NA	4.8	NA	6.7	3.5	4.7
Herpud1	7.2	12.5	13.7	NA	NA	13.7	12.6	10.8	12.9	14.0	NA	15.9	NA	NA
Hnrpdl	8.5	NA	4.3	NA	NA	6.3	6.6	2.9	NA	7.3	10.3	8.0	NA	NA
Hsp110	19.0	16.5	16.8	17.2	NA	NA	18.3	NA	17.4	18.2	NA	21.0	NA	NA
Hspa8	NA	14.9	14.7	17.3	NA	NA	17.9	17.3	17.3	19.2	NA	20.3	15.0	NA
Inmt	17.2	NA	11.2	12.2	NA	NA	14.5	13.0	14.7	12.2	NA	NA	9.5	8.5
Litaf	NA	0.2	1.5	1.2	NA	NA	1.8	0.3	1.8	NA	NA	5.3	23.8	NA
Marcks	23.8	3.4	NA	2.5	NA	NA	1.6	NA	2.3	1.6	NA	NA	23.8	23.3
Mid1ip1	NA	NA	NA	NA	13.8	13.3	17.2	18.5	19.8	NA	NA	6.0	11.2	11.7
Nedd4l	21.5	22.2	NA	9.3	NA	21.5	5.7	5.2	7.5	NA	14.8	17.5	NA	NA
Nfil3	NA	22.2	18.8	20.3	20.2	21.0	18.5	20.3	20.0	19.0	NA	23.6	20.5	19.8
Nr1d1	4.2	6.8	NA	5.7	4.5	7.5	7.5	6.5	NA	5.2	NA	9.8	5.3	5.5
Nr1d2	5.2	9.4	8.2	9.3	10.5	10.3	9.8	8.9	9.2	10.9	12.7	13.0	7.5	9.2
Pbef1	NA	12.5	11.2	14.0	NA	NA	17.7	14.7	16.2	14.8	NA	NA	13.2	NA
Pdcd4	6.4	2.7	7.2	8.2	NA	NA	4.8	8.9	NA	7.2	NA	NA	6.0	6.0
Per1	6.5	12.7	NA	NA	NA	11.5	10.5	10.8	NA	11.2	14.5	NA	12.8	11.5
Per2	8.7	15.2	13.5	13.5	13.3	13.0	13.6	12.7	13.5	12.7	14.5	16.4	12.0	12.0
Per3	NA	NA	NA	NA	NA	NA	12.8	11.0	11.5	10.8	14.1	12.3	9.6	9.5
Pim3	NA	11.9	12.2	7.0	NA	NA	7.6	6.1	6.7	4.0	NA	NA	8.7	9.2
Por	NA	13.3	17.7	9.8	NA	NA	12.3	10.0	10.5	11.3	NA	13.1	NA	NA
Rorc	NA	18.6	17.5	NA	NA	19.7	19.3	17.8	17.7	17.8	NA	NA	17.8	18.3
Tef	NA	11.4	NA	10.8	NA	13.2	11.9	9.6	11.1	10.7	11.7	14.3	10.5	11.3
Tfrc	NA	17.0	NA	NA	20.8	4.8	15.0	19.7	21.3	19.3	NA	8.2	NA	NA
Timp3	NA	NA	NA	15.8	14.5	11.7	12.1	12.2	13.3	9.3	11.8	14.7	12.0	12.0
Tsc22d3	NA	17.9	NA	NA	18.3	16.7	14.8	15.3	14.8	14.8	13.3	15.4	NA	NA
Tspan4	NA	15.7	12.2	NA	12.3	11.8	11.7	NA	12.3	10.7	NA	10.6	10.7	10.3
Usp2	NA	12.2	13.0	12.2	NA	11.7	11.4	13.1	14.0	11.7	NA	11.0	10.3	11.0

Tissue symbols: SCN, Superchiasmatic Nucleus; LIV, Liver; KID, Kidney; AOR, Aorta; SKM, Skeletal muscle; HAT, Heart; ADG, Adrenal Gland; BAT, Brown Adipose Tissue; WAT, White Adipose Tissue; BON, Calvarial Bone; PFR, Prefrontal Cortex; WB, Whole Brain; ATR, Atrium; VEN, Ventricle. NA denotes no evidence for circadian oscillation.

### Comparison between Tissues

We surveyed tissue-specific gene expression profiles in a mouse tissue gene expression atlas [7] for the circadian oscillating genes in different tissues. To cross-validate the circadian phase data with the tissue gene expression data, we created a binary matrix of 1 or 0 to denote the presence or absence of circadian oscillations in 14 tissues in circadian phase data and compared it to the gene expression matrix in 61 tissues from the tissue gene expression atlas. For each pair of tissues from the two matrices, we calculated a correlation coefficient. The circadian data in liver, kidney, skeletal muscle, adrenal gland, and white adipose tissue correctly correlated best with their corresponding tissues in the tissue gene expression atlas, whereas SCN correlated equally well with preoptic and hypothalamus, and brown adipose tissue correlated equally well with adipose tissue and brown fat. These results reflected the fact that sufficiently high gene expression levels are the prerequisite to be detected as circadian oscillating in our collection of microarray datasets.

To investigate if the differences in the circadian phases of circadian oscillating genes across tissues are caused by the differences in their gene expression levels, we calculated the variances of circadian phases and the variances of gene expression for circadian oscillating genes across the seven tissues common to our circadian datasets and the tissue gene expression atlas. There is no significant correlation (*r* = 0.01, *p* = 0.71) between these two variances. For example, the gene expression level of *Per2* is 27 times higher in adrenal gland than in skeletal muscle, but this has no effect on the consistency of circadian phases of *Per2* between the two tissues. In fact, the common circadian genes have significantly higher variances of gene expression across the 61 tissues than those from the same number of randomly selected genes. We observed that the correlation coefficients *r_ij_* between the tissue gene expression data of the common circadian gene pairs (*i*,*j*) negatively correlated with their circadian phase differences (*r* = −0.22, *p<*10^−8^). The gene pairs positively correlated in their tissue gene expression patterns had a significantly lower circadian phase difference than expected by random, whereas the gene pairs negatively correlated in their tissue gene expression patterns had a significantly larger circadian phase difference than expected by random (Figure S1). Therefore, the common circadian genes with similar gene expression patterns across tissues also tend to have similar circadian phases. The circadian gene regulation may share a similar mechanism that gives rise to tissue-specific gene expression.

**Figure 2:**
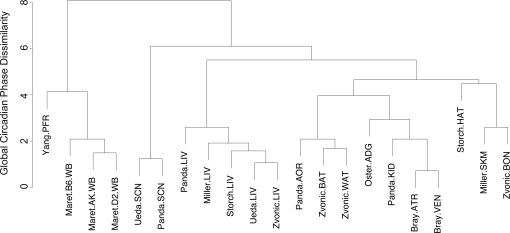
Hierarchical clustering of 21 circadian microarray datasets based on global circadian phase dissimilarities. Datasets are denoted by first author names and tissue types.

**Figure 3:**
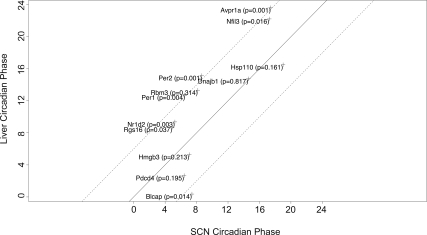
Comparison of circadian phases between SCN and liver. *p*-values from the circular ANOVA test are indicated in the parenthesis. The solid line represents *y* = *x*. The dashed lines represent *y* = *x*±6 respectively.

We clustered the 21 circadian phase datasets using hierarchical clustering. The datasets from the same tissue or biologically closely related tissues were clustered together, suggesting that the differences in circadian phases between tissues resulted from their biological differences (Figure 2). To ensure that these differences between tissues were also reproducible between experiments, we used circular ANOVA to identify the circadian oscillating genes shared between two tissues but associated with significantly different circadian phases between these tissues. There were 12 circadian oscillating genes shared between two SCN datasets and at least two liver datasets. Among them, *Per1*, *Per2*, *Nr1d2*, and *Avpr1a* showed a significant (*p<*0.01) advance of about 6 hours in their circadian phases in SCN datasets compared to liver datasets, whereas *Dnajb1*, *Hmgb3*, *Hsp110*, and *Pdcd4* showed no significant differences in their circadian phases between SCN and liver (Figure 3). To test if such differences also exist between SCN and whole brain tissues, we also compared SCN with 3 whole brain datasets. There were 12 circadian oscillating genes shared between two SCN datasets and at least two whole brain datasets. *Per2*, *Nr1d2*, and *Tuba8* again showed a significant advance of about 6 hours in their circadian phases in SCN datasets compared to whole brain datasets, whereas *Hmgb3*, *Hsp110*, *Sgk*, and *Fabp7* showed no significant differences in their circadian phases between SCN and whole brain. Further examination validated that the known key circadian genes including *Per1*, *Per2*, *Cry1*, *Arntl*, *Nr1d1*, and *Nr1d2* all showed around 6 hour advances in circadian phases between SCN and non-SCN tissues in general, whereas heat shock proteins showed consistent circadian phases across all tissues. There were 15 circadian oscillating genes shared between 3 heart datasets including whole heart, atria, and ventricle and at least 3 liver datasets. Comparing the heart datasets with the liver datasets, *Bhlhb2* (*p<*0.001) and *Tspan4* (*p* = 0.006) had circadian phase 5–6 hours earlier in heart than liver whereas *Dscr1* (*p* = 0.002) had circadian phase 8 hours later in heart than liver. Other known key circadian genes such as *Per1*/*Per2*, *Arntl*, and *Nr1d1*/*Nr1d2* showed consistent circadian phases between heart and liver. Comparing the whole brain datasets with the liver datasets, *Tfrc*, *St3gal5*, and *Tspan4* had circadian phases more than 4 hours earlier in whole brain than liver, whereas *Hist1h1c*, *Tsc22d1*, *Myo1b*, *Litaf*, and *BC004004* had circadian phases more than 4 hours later in whole brain than liver.

### Comparison between Mammalian Species

Among the 1,269 rat genes identified as circadian oscillating genes in rat liver, 1,137 of them had homologues in mouse. 232 of them overlapped with 944 mouse liver circadian oscillating genes in at least 2 mouse liver datasets. We used the circular ANOVA test to identify the circadian oscillating genes shared in both mouse and rat livers but with significantly different circadian phases. 10 genes had significantly (*p*<0.01) different circadian phases between mouse and rat livers. The circadian phases of *BC006779*, *Cdkn1a*, *Svil*, *Uox*, *Ak2*, *Nr1d1*, *Mtss1*, *Nudt16l1*, and *Gss* were 4–6 hours later in rat liver than mouse liver, whereas *Hsd17b2* was in anti-phase between mouse and rat livers (Figure S2).

Among 803 rat skeletal muscle (SKM) circadian oscillating genes, 703 of them had homologues in mouse and 64 of them overlapped with 440 mouse SKM circadian oscillating genes. Among the overlapping genes, 34 of them did not show circadian phase differences larger than 4 hours between mouse and rat SKM. 22 of them had circadian phases more than 4 hours later in rat SKM than mouse SKM. *Cpt1a*, *Pdk4*, and *Ucp3*, involved in lipid metabolism, showed a 5–8 hour delay in their circadian phases in rat SKM compared to mouse SKM. 8 genes had circadian phases more than 4 hours earlier in rat SKM than in mouse SKM. Among them, *Fkbp5* and *Sgk*, which are controlled by the glucocorticoid receptor element (GRE), had about 6 hour advance in their circadian phases in rat SKM compared to mouse SKM. There were 11 circadian oscillating genes common to mouse liver and SKM, and rat liver and SKM. The 4–5 hour delay in circadian phases in rat compared to mouse was observed in both liver and SKM for all 11 circadian genes except *Dynll1*.

Among 603 rhesus macaque adrenal gland circadian oscillating genes, 560 had homologues in mouse and 170 overlapped with 4,162 mouse adrenal gland circadian oscillating genes. We found significant differences in circadian phases also between these two species. Among the overlapping genes, 47 did not show circadian phase differences larger than 4 hours between mouse and macaque, whereas 66 had circadian phases more than 4 hours later in the macaque adrenal than in the mouse adrenal. Known key circadian genes, *Arntl*, *Dbp*, *Nr1d1*, and *Bhlhb2*, showed about 8 hour delay in their circadian phases in the macaque adrenal compared to the mouse adrenal. Although *Per2* did not satisfy our criteria (*p*<0.01) to be a circadian oscillating gene in macaque adrenal, this gene has a circadian phase at CT21 (*p* = 0.03), which is also about 8 hours later than that in mouse. Similarly, heat shock proteins, *Hsp110*, *Hspa8*, *Dnaja1*, and *Dnajb6*, had circadian phases around CT16 in the mouse adrenal but around CT0 in the macaque adrenal. Cold inducible protein (*Cirbp*) had a circadian phase around CT7 in the mouse adrenal but around CT16 in the macaque adrenal, in anti-phase with heat shock proteins in both mouse and macaque. On the other hand, there were also 57 genes showing circadian phases more than 4 hours early in the macaque adrenal than in the mouse adrenal.

In the human circadian SKM microarray study, there were only two circadian time point measurements: CT1 and CT13. Hence we can only roughly estimate the circadian phases to be either CT1 or CT13 in human SKM. Among the common circadian genes, *Per1*, *Per2*, *Nr1d2*, and *Dbp* had circadian phases around CT1, whereas *Arntl* and *Cry1* had circadian phases around CT13 in human SKM. Our estimates of circadian phases for *Per1* and *Per2* in human SKM were in good agreement with the study in human peripheral blood mononuclear cells where a 2 hour sampling time was used throughout 72 hours [8]. The heat shock proteins, *Dnaja1*, *Dnajb4*, and *Hspa4*, had circadian phases around CT13, consistent with the peak of common body temperature at CT10 in human [8].

Next, we made a three-species comparison of circadian phases in the SKMs of mouse, rat, and human. We found 12 circadian oscillating genes common to SKM in all three species (Table 2). After we rounded the circadian phases in mouse and rat to their closest time points, CT1 or CT13, we observed that *Per2*, *Arntl*, *Dbp*, *Ppp1r3c*, and *Ablim1* had conserved circadian phases between mouse and rat, but were 12 hours away from those of human. *Epm2aip1*, *G0S2*, and *Maf* had conserved circadian phases between mouse and human but 12 hours away from those of rat. Finally, *D19Wsu162e*, *Myod1*, *Pfn2*, and *Ucp3* had conserved circadian phases among all three species.

**Table 2:** Circadian oscillating genes common to the SKMs of mouse, rat, and human.

Gene Symbol	Mouse SKM	Rat SKM	Human SKM
Ablim1	19.00	0.83	13
Arntl	23.00	2.33	13
D19Wsu162e	21.17	23.00	1
Dbp	10.00	12.33	1
Epm2aip1	13.33	19.67	13
G0s2	16.33	21.83	13
Maf	14.67	5.17	13
Myod1	16.67	18.83	13
Per2	13.33	16.00	1
Pfn2	14.50	18.08	13
Ppp1r3c	21.50	23.33	13
Ucp3	0.17	5.50	1

### Biological Functions of the Circadian Rhythm

We searched for the Gene Ontology (GO) categories significantly over-represented in circadian oscillating genes in each mouse tissue using GOminer program [9]. We further tested the associations of GO categories with any specific circadian phase intervals using Fisher's test with a rotating window method. The list of significant biological processes associated with circadian phases in different tissues is shown in Table S3. The most common of these biological processes were steroid biosynthesis, heat shock response, and protein folding. Steroid biosynthesis was associated with CT22 in liver, kidney, adrenal, brown adipose tissue (BAT), and white adipose tissue (WAT). Heat shock response or protein folding were associated with CT16 in SCN, liver, kidney, adrenal, aorta, BAT, WAT, calvarial bone, and whole brain, due to a large number of heat shock proteins consistently showing circadian phases near CT16 in most tissues. In liver, carbohydrate and amino acid metabolism were associated with CT17 and CT15 respectively, consistent with the rise of activities after light off in mouse. In BAT, WAT, and adrenal, lipid metabolism was associated with CT22. Negative regulation of protein kinase activities was associated with CT17 in prefrontal cortex and CT21 in whole brain. There were also notable differences in the circadian phases of some biological processes between tissues. For example, protein translation was associated with CT20 in SCN but CT9 in WAT. Organ development was associated with CT22 in heart and BAT but CT10 in adrenal.

### Promoter Analysis

To test the association of transcription factor (TF) regulation with the circadian oscillation of gene expression, we predicted the TF binding sites on the mouse promoters of circadian oscillating genes in each tissue using positional weight matrix (PWM) based methods. We first tested whether there was a significant over-representation of TF PWM binding sites on the promoters of circadian oscillating genes using the Fisher's exact test. Among the significant TF PWMs, we again tested their associations with any specific phase intervals using the Fisher's test with a rotating window method. To remove the redundancy in TF PWMs, we grouped the TF PWMs into TF families and averaged the associated circadian phases of significant TF PWMs within the same TF families. The results are shown in Table S4. EBOX, AP-2, CRE, SP1, and EGR were the top 5 TF families associated the circadian phase in most tissues. However, unlike the consistent circadian phases of the common circadian genes across tissues, the associated circadian phases of the significant TF families varied considerably among different tissues. EBOX was associated with CT12 in the majority of tissues including SCN, liver, aorta, adrenal, WAT, brain, atria, ventricle, and prefrontal cortex, but it was associated with CT0 in skeletal muscle, BAT, and calvarial bone. CRE was consistently associated with CT11 in SCN, liver, aorta, heart, adrenal, calvarial bone, prefrontal cortex, and ventricle, but with CT20 in atria. Two other known TF families related to circadian rhythm, RRE and DBOX, were detected to be associated with circadian phase only in two tissues. RRE was associated with CT0 in liver and WAT. DBOX was associated with CT16 in aorta and adrenal.

### Identification of Gene Regulatory Interactions

We obtained microarray data from TF knockout or mutants for *Clock*, *Arntl*, *Npas2*, *Nr1d1*, *Rora*/*Rorc*, *Egr1*/*Egr3*, *Dbp*/*Hlf*/*Tef*, and *Ppara* in various mouse tissues, together with *Cebpa*/*Cebpb*/*Cebpd*/*Cebpe* transfection microarray data in NIH3T3 cells. To study the systematic effects of glucocorticoids, cAMP, and temperature on the circadian rhythm, we included microarray data from *Nr3c1* (glucocorticoid receptor), *Pka*, and *Hsf1* knockouts or mutants in response to DEX (glucocorticoid agonist), cAMP, and heat stimulation, respectively, compared with wild type mouse. We also included microarray data from a light response mouse model in order to identify light sensitive genes in mouse SCN [10]. The complete list of knockout or mutant microarray experiments used in this study is shown in Table S5. We assumed that the target genes of TFs will be significantly down-regulated in the knockout or mutant compared with the wild type mouse in the case of activators, and up-regulated in the case of repressors, such as *Nr1d1*. To identify the direct targets of TFs in knockout or mutant experiments, we required that the significantly affected genes in the knockout or mutant must have at least one putative binding site of their corresponding TFs in the promoter regions. Under these criteria, we identified 320 EBOX, 295 RRE, 43 DBOX, 492 EGRE, 455 CRE, 326 GRE, 122 HSE, 607 CEBP, and 516 PPRE controlled genes respectively (Table S6). For these genes, we extracted their mean circadian phases if they have consistent circadian phases across multiple tissues (*p*<1/3, circular range test). We observed that EBOX was significantly associated with CT12 (*p*<10^−6^, Fisher's exact test), RRE with CT1 (*p*<10^−6^), DBOX with CT15 (*p*<10^−5^), HSE with CT17 (*p*<10^−6^) (Figure S3).

### Circadian Gene Regulatory Network

**Figure 4:**
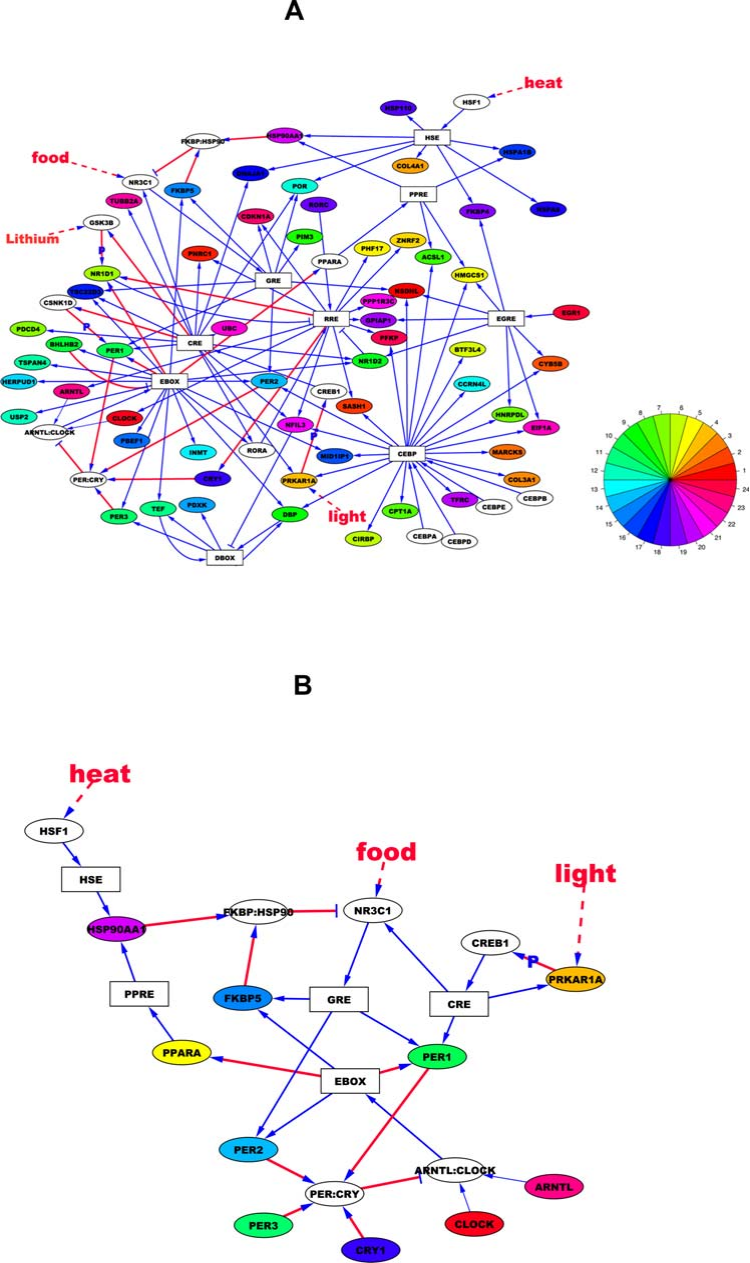
Circadian gene regulatory network in mouse. (A) Gene regulatory network consisting of the circadian oscillating genes identified in at least 7 mouse tissues. (B) The subset of network highlighting NR3C1 and FKBP/HSP90's role of integrating the regulatory inputs from diverse environmental signals into circadian genes. Blue arrows represent the gene regulatory interactions obtained in this study. Red arrows represent the known gene regulatory or protein interactions extracted from the literature. P stands for phosphorylation. White boxes represent *cis-*regulatory elements. Colored circles represent the genes with circadian phase information, where circadian phases are represented by the different colors in the color wheel. White circles represent protein complexes or genes without circadian phase information.

Based on these regulatory interactions, we constructed the gene regulatory network for the circadian oscillating genes in mouse. In Figure 4, we show a network consisting of the circadian oscillating genes identified in at least 7 mouse tissues. Among the 81 circadian oscillating genes identified in at least 7 tissues, 53 of them can be included through 88 regulatory interactions with 9 *cis-*regulatory elements in our network. Their circadian phases were represented by different colors in the color wheel. We were able to identify almost all known transcription regulatory interactions for common circadian genes in the literature, except EBOX → Per1, EBOX → Nr1d1, EBOX → Ppara, RRE → Nr1d1, and RRE → Cry1. To further complete our network, we supplemented these missing gene regulatory interactions with known protein interaction information (Per/Cry 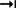 Arntl/Clock and Fkbp:Hsp90 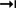 Nr3c1) and protein phosphorylation information (Csnk1d → Per/Cry and Gsk3b → Nr1d1) from the literature. These relationships are shown in red color in Figure 4.

Two well-known negative feedback loops can be reconstructed from this analysis: Arntl/Clock → EBOX → Per1/Per2 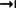
Arntl/Clock and Nr1d1/Nr1d2 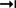 RRE → Arntl/Clock → EBOX → Nr1d1/Nr1d2. Two feedforward loops are attached to the negative feedback loops through Arntl/Clock → EBOX → Dbp → DBOX → Per1/Per2 acting as an alternative route of Arntl/Clock → EBOX → Per1/Per2 and Nr1d1/Nr1d2 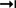 RRE → Nfil3 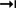 DBOX → Per1/Per2 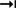 Arntl/Clock acting as an alternative route of Nr1d1/Nr1d2 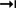 RRE → Arntl/Clock. Bhlhb2 inhibiting EBOX is also regulated by EBOX and Nr1d1 inhibiting RRE is also regulated by RRE, therefore forming two auto-regulatory loops.

The effects of food and light act on common circadian genes directly through GRE and CRE respectively. GRE controls Per1 and Per2, while CRE controls Per1, Rora, Nr1d2, and Nfil3. As shown in Figure 4B, the effect of temperature acts on common circadian genes rather indirectly through the route HSE → Hsp90aa1 → Fkbp/Hsp90 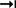 Nr3c1 → GRE → Per1/Per2. Nr3c1 and the Fkbp/Hsp90 complex are also components of another negative feedback loop, Nr3c1 → GRE → Fkbp5 → Fkbp/Hsp90 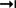 Nr3c1, which may play an important role in glucocorticoid stimulation. Nr3c1 is also under the control of CRE and therefore may be responsive to light stimulation. Nr3c1 and the Fkbp/Hsp90 complex feed into EBOX by regulating Per1/Per2 through GRE. In turn, EBOX controls both components of the Fkbp/Hsp90 complex, i.e., Fkbp5 directly and Hsp90aa1 indirectly through EBOX → Ppara → PPRE → Hsp90aa1. Therefore, Nr3c1 and Fkbp/Hsp90 play central role of integrating the regulatory inputs from diverse environmental signals into circadian genes in our network (Figure 4B).

## Discussion

By combining all available circadian microarray data in mouse, we identified a set of common circadian genes showing circadian oscillations with consistent circadian phases in a wide range of tissues. However, the majority of circadian oscillating genes were restricted to a small number of tissues, with large variations in their circadian oscillation phases, suggesting that they are likely circadian-controlled genes that are driven by common circadian genes under their different tissue environments. The 6 hour phase delay of known key circadian genes such as *Per1*, *Per2*, and *Nr1d1* in non-SCN tissues compared to SCN has been noted by others previously and has been explained by the time-lapse needed to transmit the regulatory signals from SCN to peripheral tissues. However, we also observe genes such as heat shock proteins showing consistent phases in all tissues including SCN, which coincide with the phase of circadian oscillation of body temperature in mouse. The circadian oscillation of body temperature may hence be the driving force that synchronizes the circadian oscillation of heat shock proteins throughout the body, which may be independent of the regulation of circadian rhythm in peripheral tissues by SCN.

After integrating tissue gene expression data with circadian rhythm data, we were surprised to find that the common circadian genes show a high degree of variation in gene expression across tissues in spite of the universal presence of circadian rhythms in different tissues. This indicates that the circadian rhythm gene regulatory network is robust against the variations in gene expression levels of its key components in different tissues.

Interestingly, we observed that the common circadian genes with similar gene expression patterns across tissues also tended to have similar circadian phases. Thus, the gene regulatory network responsible for generating “spatial” expression variation across tissues may be also responsible for generating the “temporal” expression variation.

We applied promoter analysis on the circadian oscillating genes in different mouse tissues and identified a suite of transcription factors that potentially play important roles in circadian rhythm. Bozek et al. used a similar promoter analysis approach on several mouse circadian microarray datasets and identified TFs including Sp1, AP2, STAT1, HIF-1, and E2F to be associated with circadian oscillating genes [6]. However, they considered neither tissue differences nor the association of TFs with specific circadian phases. Furthermore, using sequence based promoter analysis alone to identify significant TFs that regulate circadian oscillating genes is problematic. First, it is almost impossible to distinguish the multiple TFs binding to identical or similar DNA motifs. For example, in addition to *Arntl*/*Clock*, a number of other TFs such as *Usf* and *c-myc* also bind the EBOX motif. Second, it is difficult to separate the direct and indirect regulatory interactions. For example, although we identified the association of TFs such as SP1, E2F, and A2P with circadian oscillating genes, it is more likely that these TFs are associated with other key circadian TFs such as Arntl/Clock, and act as parts of the transcription machinery. To overcome these problems, we utilized a number of mouse TF knockout or mutant microarray experiments to construct a systematic gene regulatory network for circadian rhythm in mouse. We compared our network with a small-scale gene regulatory network constructed by Ueda et al. using a reporter assay for 16 common circadian genes in mouse [5]. Among the nine E/E'BOX controlled genes identified by Ueda et al., *Per1*, *Per2*, *Bhlhb2*, *Bhlhb3*, *Cry1*, *Dbp*, *Nr1d1*, *Nr1d2*, and *Rorc*, we identified five, *Per2*, *Per3*, *Bhlhb2*, *Dbp*, *Nr1d2*, and also *Rora* instead of *Rorc*. Among the seven DBOX controlled genes identified by Ueda et al., *Nr1d1*, *Nr1d2*, *Rora*, *Rorb*, *Per1*, *Per2*, and *Per3*, we only identified *Per3*. Among the six RRE controlled genes identified by Ueda et al., *Clock*, *Npas2*, *Arntl*, *Nfil3*, *Rorc*, and *Cry1*, we identified four, with *Rorc* and *Cry1* being the exceptions. In fact, *Cry1* was significantly up-regulated in the *Nr1d1* knockout experiment, but we did not identify any canonical RRE binding site in its promoter, suggesting our criterion for putative RRE may be too stringent. Ueda et al. showed that the transcriptional activities of EBOX, RRE, and DBOX reach their maximums at CT7.5–CT11.5, CT21.0–CT23.0, and CT11.0, respectively. The circadian phases associated with EBOX and RRE in our network were consistent with Ueda et al.'s results whereas the circadian phase associated with DBOX was around CT15–CT16 in our network.

An important question in circadian physiology is how environmental factors such as food, light, and temperature affect the circadian clock. Upon food intake, adrenal gland secretes glucocorticoids that activate the glucocorticoid receptor (*Nr3c1*). It was known that the activated *Nr3c1* positively regulates *Per1* through a glucocorticoid responsive element (GRE) in the *Per1* promoter. Here we show that the direct targets of *Nr3c1* also include other common circadian genes such as *Per2* and *Fkbp5*.

Upon cAMP stimulation, PKA phosphorylates CREB1, which in turn up-regulates downstream genes through the cAMP responsive element (CRE). One component of PKA, *Prkar1a*, was among the common circadian genes that we identified with a phase at CT2.5. Other components of PKA were also found to be oscillating with phases around CT0. The rhythmic oscillation of the mRNA levels of PKA components may suggest that the cAMP signaling pathway is circadian oscillating even in the absence of light stimulation, as many microarray experiments were conducted in 12 h dark:12 h dark (DD) condition. It is known that the *Per1* promoter contains a functional CRE responsive to cAMP stimulation. Our analysis of PKA mutant microarray data identified additional CRE controlled common circadian genes such as *Nr1d2*, *Nfil3*, and *Rora*. In addition, CRE also controls two kinases, *Csnk1d* and *Gsk3b*, playing important roles in post-transcriptional regulation of common circadian genes. *Csnk1d* is a key kinase that phosphorylates PER1 proteins in the cytoplasm, which leads to their degradation. Thus, cAMP stimulation not only elevates the mRNA levels of *Per1*, but also the phosphorylation state of PER1 proteins in the cytoplasm. *Gsk3b* has been shown to phosphorylate and stabilize *Nr1d1* protein. The inhibition of *Gsk3b* activities by lithium has also been implicated in the treatment of bipolar and circadian disorders [11]. In mouse, the response to light has long been suggested to be acting through the cAMP signaling pathway. We identified 28 light sensitive genes in mouse SCN from the light response microarray experiment. Seven of them are PKA controlled genes that we identified from PKA knockout experiments. There are only two genes, *Egr1* and *Pim3*, among the common circadian genes. They were not among the CRE controlled genes identified from PKA knockout experiments. But a closer examination showed that both genes have conserved CREs between human and mouse in their promoters, therefore strongly suggesting that they too were controlled by CRE.

As a key TF in heat response, *Hsf1* mainly controls heat shock proteins, whose circadian phases are significantly enriched around CT16, coinciding with the phase of daily body temperature oscillation in mouse. *Hsp90aa1* is a direct target of *Hsf1*. *Fkbp5* and *Hsp90* form a complex inactive glucocorticoid receptor and transmit the impact of heat stimulation indirectly on *Per1*/*Per2*. Kornmann et al. suggested that temperature might entrain the circadian rhythm through the direct regulation of *Hsf1*/*Hsf2* on *Per2* [12]. However, we found no evidence of such direct regulation either from the *Hsf1* knockout experiment or from the *Per2* promoter analysis. Instead, our result suggests an indirect regulation of *Hsf1* on *Per2* through the glucocorticoid receptor. Similar crosstalk between glucocorticoid stimulation and cAMP stimulation may also exist, as our results showed that the promoter of glucocorticoid receptor *Nr3c1* also contained CRE and was responsive to cAMP signaling. *Cebp* family proteins have a significant number of inputs to common circadian rhythm genes such as *Per2*, *Dbp*, and *Nfil3*. *Cebpa* showed circadian phase at CT7 in four tissues, *Cebpb* at CT11 in six tissues, and *Cebpd* at CT14 in two tissues. Their circadian phases suggest that they may be driven by *Arntl*/*Clock* through EBOX, thereby forming additional feedback loops. *Npas2* has been considered to be a substitute for *Clock* in forming a hetero-dimer with *Arntl*. We only obtained 47 *Npas2* regulated genes from *Npas2* knockout experiment and only one gene, *Cirbp*, was among the common circadian gene. Therefore, *Arntl*/*Npas2* may have only played a minor role in circadian rhythm comparing to *Arntl*/*Clock*.

Metabolism and cell cycle are among the many important biological processes controlled by the circadian rhythm. *Pfkp*, a key enzyme which controls glycolysis and shows circadian phase around CT23 in 7 tissues, is regulated by RRE. *Ces3*, a key enzyme in fatty acid metabolism showing circadian phase around CT17 in 6 tissues, is controlled by DBOX. *Ppara*, a key TF regulating fatty acid metabolism showing circadian phase around CT7 in three tissues, is controlled by EBOX and may drive the circadian oscillation of other downstream metabolic genes. The circadian oscillations in the cAMP signaling pathway as discussed earlier will also undoubtedly affect the metabolism. In liver, the main metabolic organ, carbohydrate and amino acid metabolism, were associated with CT17 and CT15 respectively. In adipose tissues such as BAT and WAT, lipid metabolism was associated with CT22. We also observed the association of CT0 with steroid biosynthesis in a wide range of tissues. These results are consistent with the observation that the metabolic activities rise after light off (dusk) in mouse.

*Cdkn1a* or *p21*, a cyclin dependent kinase inhibitor controlling the progression of cell cycle at G1 phase has the circadian phase at CT22 in 10 tissues and is controlled by RRE. Another kinase, *Wee1*, controlling the progression of cell cycle into M phase, has circadian phase at CT14 in 5 tissues and is controlled by DBOX. *Cdkn1a* and *Wee1* are two valves controlling the G2/M and G1/S checkpoints in cell cycle progression, respectively. They have almost opposite circadian phases and receive inputs from the negative limb *Nr1d1* and the positive limb *Dbp* in the circadian rhythm, respectively, which leads to the orchestrated progression of the cell cycle by circadian clock.

The mouse has been the most extensively studied mammalian model organism for circadian rhythm. The scarcity of microarray experiments with circadian and TF knockouts or mutants in non-mouse mammals makes it difficult to construct systematic gene regulatory networks for non-mouse mammals. But the comparison between the microarray experiments in mouse and a few microarray experiments in other mammals including rat, macaque, and human, have revealed significant differences between species both in terms of circadian oscillating genes and their circadian phases. The known key circadian genes showed a 4–5 hour phase delay in rat compared to mouse and 8–12 hours phase delay in macaque and human compared to mouse, which probably reflects the fact that mouse and rat are nocturnal animals whereas macaque and human are diurnal. Interestingly, the circadian phases of heat shock proteins are well aligned with the peaks of body temperature in mouse, rat, and human. The anti-phase relationship between EBOX controlled genes and RRE controlled genes is preserved among mouse, rat, macaque, and human. Therefore, the negative feedback loops in the center of the mammalian circadian rhythm, consisting of *Per1*/*Per2*, *Cry1*, *Arntl*, *Clock*, and *Nr1d1*/*Nr1d2*, must have been well conserved among mammalian species. Meanwhile, the diversity in the circadian oscillating genes and their phases among these four species suggests that a significant amount of gene regulatory interactions in the circadian gene regulatory network have been rewired during evolution. Future comprehensive studies on the structure and dynamics of circadian gene regulatory networks in different mammalian species will advance our understanding of the molecular basis of their physiological and behavioral differences.

## Materials and Methods

### Circadian Microarray Data

We collected all available circadian microarray data from different laboratories for mouse, rat, rhesus macaque (*Macaca mulatta*), and human. The total mouse data consisted of 21 datasets covering 14 tissues including two datasets in SCN, five datasets in liver, three datasets in whole brain, one dataset in kidney, aorta, heart, skeletal muscle (SKM), adrenal gland, brown adipose tissue (BAT), white adipose tissue (WAT), calvarial bone, prefrontal cortex, atria, and ventricle. The three datasets in whole brain were from three different mouse strains: C57BL/6J, AKR/J, and DBA/2J. The rat data consisted of one dataset in liver and one dataset in skeletal muscle. The macaque data consisted of one dataset in adrenal gland. The human data consisted of one dataset in skeletal muscle. The complete list of all circadian microarray datasets used in this study is shown in Table S1. Most circadian microarray experiments were conducted in a time series of every 4 hours. The human microarray experiment was only conducted at CT1 and CT13. For simplicity, we did not distinguish the light conditions, i.e., 12 h light:12 h dark (LD) or 12 h dark:12 h dark (DD), under which the animals were kept during the experiments. In order to have a more complete and consistent analysis of the data from different experiments, we decided to re-analyze all the datasets by our own method rather than simply taking the gene lists from the original publications. For the datasets where the CEL files were available, we normalized the data by RMA method in “affy” package. For the datasets where only normalized data were available, the normalization step was skipped.

We used the method similar to that described in [2] to analyze all microarray data. Namely, cosine functions *A_ij_*(*t*) = cos(2π*t*/*T_i_*−*ϕ_j_*) where *T_i_* = 20+*i*, *ϕ_j_* = 2π*j*/60, 0 ≤ *i* ≤8, and 0 ≤ *j* ≤59 were used as the reference time series of circadian oscillation. The gene expression time series of each probe set on the microarray were fitted to each cosine function time series *A_ij_*(*t*) and the cosine function with highest correlation coefficient was chosen. A *p*-value <0.01 in the regression for the best cosine function was used as the criterion for circadian oscillation, and we estimated a false positive rate of about 10% for this cutoff using a random permutation test. When the experimental replicas at each time point were available, we further carried out a one-way ANOVA test on the time series using time points as factor and *p*-value <0.05 as an additional criterion. For the probe sets satisfying the criteria for circadian oscillation, the gene expression time series were again fitted to the cosine functions with fixed 24 hrs period but changing phases, *B_j_*(*t*) = cos(2π*t*/*T*−*ϕ_j_*), where *T* = 24, *ϕ_j_* = 2π*j*/144, and 0≤ *j* ≤ 143. The circadian phase was calculated from the best fitted *B_j_*(*t*) as ϕ_j__*_24/2π. We were unable to obtain the microarray data in [2] so we only extracted circadian gene lists with their circadian phase information. In the human SKM study, vastus lateralis muscles were taken from exercised and non-exercised legs of 4 patients at CT1 (8AM) and CT13 (8PM). We used circadian time and exercise state as two factors in two-way ANOVA. A *p*-value <0.05 in the circadian time comparison was used as the criterion for circadian oscillation. We estimated the circadian phase to be either CT1 or CT13, depending on when the average expression value was the highest in human SKM.

The R package “Circular” was used to analyze the circadian phases obtained from circadian microarray datasets. For each circadian microarray dataset, the probe sets were annotated by R package “annaffy” and only the probe sets corresponding to known genes were used in the analysis. The probe sets that passed circadian oscillation criteria and that corresponded to the same genes were merged by the following procedure. First, a circular range test was used to assess the consistency of phases estimated from the different probe sets for the same genes, where *p*<1/3 was used as the criterion to take into account the 4 hour intrinsic errors in phase estimation as the animals were sampled every 4 hours in most experiments. Then, a circular mean function was used to calculate the mean circadian phases from the consistent probe sets. The same procedure was used to combine the different datasets for the same tissue, i.e., five datasets for liver, two datasets for SCN, three datasets for whole brain. In liver and whole brain, we only selected the genes identified as circadian oscillating in at least two out of five liver datasets or two out of three whole brain datasets, respectively. In SCN, we selected the genes identified as circadian oscillating in one out of two SCN datasets considering the small number of circadian oscillating genes in Ueda et al.'s SCN dataset [2]. We identified 9,955 circadian oscillating genes in at least one out of 14 tissues (Table S2). The number of circadian oscillating genes in different number of tissues was plotted using the “barplot” function in R and is shown in Figure 1A. The circular range test was also used to describe the consistency of phases of circadian oscillating genes across tissues. The distribution of *p*-values of circular range tests in different number of tissues was plotted using the boxplot function in R and is shown in Figure 1B. We defined the 41 circadian oscillating genes identified in at least 8 out of 14 tissues as common circadian genes, and these are shown in Table 1.

### Tissue-Specific Gene Expression of Circadian Oscillating Genes

The microarray data of 61 mouse tissues after gcrma normalization were downloaded from the mouse tissue gene expression atlas website: http://symatlas.gnf.org [7]. We selected the probe set with the highest average expression value across tissues to represent the genes with multiple probe sets. To remove the non-detected probe sets, we filtered out the probe sets with gene expression values lower than 100 in all 61 tissues. We obtained the expression profiles for 19,168 genes across 61 tissues. For 9,955 circadian oscillating genes identified in at least one tissue, we created a matrix of 1 or 0 to denote the presence or absence of circadian oscillation in 14 tissues. For 8,029 genes having both circadian data and tissue expression data, we calculated the correlations between the circadian 1 or 0 matrix with the matrix of log_2_(gene expression) in 61 tissues in tissue gene expression atlas. We searched for the tissues in tissue data having the highest correlation coefficient with the tissues in circadian data. Liver (*r* = 0.29, *p*<10^−15^), kidney (*r* = 0.23, *p*<10^−15^), skeletal muscle (*r* = 0.10, *p<*10^−15^), adrenal gland (*r* = 0.06, *p* = 10^−7^), and white adipose tissue (*r* = 0.18, *p<*10^−15^) in circadian data have the highest correlations with their corresponding tissues in the tissue data, whereas SCN in circadian data correlates equally well with preoptic and hypothalamus (*r* = 0.22, *p*<10^−15^) in tissue data and BAT correlates equally well with adipose tissue and brown fat (*r* = 0.19, *p*<10^−15^). For the seven tissues having both circadian data and tissue data: liver, heart, BAT, WAT, kidney, adrenal gland, and SKM, we calculated the variances of circadian phases in circadian data using the “circular var” function for the circadian oscillating genes identified in at least two tissues, and the variances of log_2_(gene expression) in tissue data across the tissues where the circadian oscillations have been identified in circadian data. The correlation coefficient of these two variances is 0.01 (*p* = 0.71). For the 37 common circadian genes identified in at least 8 tissues having tissue data, the median of variances of log_2_(gene expression) across 61 tissues was 2.28. In comparison, the expected median of variances of log_2_(gene expression) for the same number of randomly selected genes was 0.54 based on 10^6^ random simulations. The correlation coefficients *r_ij_* between the tissue gene expression profiles of the common circadian gene pairs (*i*,*j*) were negatively correlated with their circadian phase differences *d_ij_* (*r* = −0.22, *p*<10^−8^). To further demonstrate the relationship between *r_ij_* and *d_ij_*, we defined two functions *y*_+_(*x*) = median(*d_ij_*(*r_ij_*>*x*)) and *y*_−_(*x*) = median(*d_ij_*(*r_ij_*<*x*)) for −1 ≤ *x* ≤ 1. We plotted *y*_+_(*x*) and *y*_−_(*x*) in Figure S1. *y*_+_(*x*) for *x*>0 is significantly lower than the median of *d_ij_* for all gene pairs (5.84) and reaches the minimum 3.068 at *x* = 0.64, whereas *y*_−_(*x*) for *x*<0 is significantly higher and reaches the maximum 9.939 at *x* = −0.26. These results indicated that the common circadian genes with positive correlations in their tissue gene expression profiles tended to have closer circadian phases, whereas those with negative correlations in tissue gene expression profiles tended to have larger differences in their circadian phases.

### Comparison between Tissues and Species

We used the median of phase differences of circadian oscillating genes shared by two tissues as the distance measure of global phase dissimilarity between two tissues. We use these distances to cluster the phases of circadian oscillating genes in all 21 datasets using hierarchical clustering with complete linkage (Figure 2). For the mouse tissues where multiple datasets were available, i.e., liver, SCN, whole brain, and heart (whole heart, atria, and ventricle), we conducted pair-wise comparisons of the phases across tissues, using the “circular ANOVA” function for the genes identified as circadian oscillating in at least two datasets in each tissue under comparison. The same method was used to compare mouse liver data with rat liver data. To compare the circadian oscillating genes across species, rat, macaque, and human gene symbols were converted to mouse orthologs using the HomoloGene database of NCBI (build 56, http://www.ncbi.nlm.nih.gov/HomoloGene).

### Gene Ontology Analysis

Gene symbols of circadian oscillating genes identified in each tissue in mouse, rat, macaque, and human were uploaded to Gominer [9] for Gene Ontology (GO) annotation and enrichment analysis. We selected the biological processes significantly over-represented in circadian oscillating genes in each tissue using False Discovery Rate (FDR) less than 0.05 as the criterion. For the circadian oscillating genes in each enriched biological process, we further tested their associations with any specific phase intervals using the Fisher's exact test with a rotating window method. In each 1,000 equally spaced phase intervals of size 4 hours between CT0 and CT24, the Fisher's test was applied to test the association between the biological process and the phase interval. The smallest *p*-value among Fisher's tests in all intervals was obtained to represent the significance of the association. The significant biological processes (*p*<0.005) in each tissue were colored using a color circle to represent their associated circadian phases. We visualized the significant biological processes as GO maps created by Cytoscape program (version 2.5). The significant biological processes were represented by the nodes and their hierarchical GO relationship was represented by the directed edges between them so that close-related biological processes were clustered together. All GO maps in different tissues can be found in our website (http://www.picb.ac.cn/circadian/). We manually selected the most representative biological processes for each GO cluster and summarized the result in Table S3.

### Promoter Analysis

Transcriptional start sites (TSSs) information of mouse and human were integrated from three databases: DataBase of Transcriptional Start Site (DBTSS) [13],[14], the CAGE (Cap-Analysis Gene Expression) database of Fantom3 (Functional annotation of mouse) project [15], and the NCBI RefSeq database [16]. The criteria to select the TSSs were as follow: for DBTSS TSSs, the proportion of confident cDNA clones (non-exonic start clones, i.e., the clones mapped to the non-exonic regions of the genome) was not less than 0.75; for CAGE TSSs, the total number of corresponding CAGE tags was not less than 2 and can be mapped around the 5′ end of a known mRNA. If no TSS can be found for the gene from either DBTSS or CAGE under the above criteria, the 5′ end of the mRNA in RefSeq (human build 36.1, mouse build 36) was used as the TSS of the gene. Human (hg18 or NCBI build36.1) and mouse (mm8 or NCBI build 36) genome sequences were downloaded from UCSC. The 3000bp flanking sequences of each TSS were extracted from the genome as the promoter regions. As the CAGE database was based upon the older versions of human and mouse genome, i.e., hg17 and mm5, we mapped the CAGE TSSs to the new version of genomes using liftOver program in UCSC. In addition, orthologous promoter regions of mouse (mm8) vs. human (hg18) genome alignment results were also downloaded from UCSC.

Three positional weight matrix (PWM) based motif searching programs, match [17], motifscan [18], and profilestas [19], were used to identify the putative transcriptional factor binding sites (TFBS) on the extracted promoter regions. All vertebrate PWMs in TRANSFAC 11.2 were used as inputs in these programs. For the match program, we used the cut-off profile that minimizes the false positive rate, i.e., minFP profile in TRANSFC 11.2. For the motifscan program, we used a third-order background model by the CreateBackgroundModel program [20] to distinguish between the motifs that occurred frequently throughout the genome and the ones that were specific to the promoter regions. For the profilestas program, we first used the profilestas package to generate the scoring matrix and scoring threshold that minimized the false positive rate for each PWM. Then we used the patser program [21] to scan the promoter sequences and select the TFBSs above the scoring thresholds. The putative TFBSs predicted from all three programs have been compared and yielded very similar results. For simplicity, all the promoter analysis results presented in this paper were based on the match program.

We first tested the significant over-representation of putative TFBSs among a total of 568 PWMs on the promoters of circadian oscillating genes using the Fisher's exact test (*p*<10^−4^), using the promoters of all known genes as the background. Among the significant PWMs, we again tested their associations with any specific circadian phase interval in each tissue using the Fisher's exact test with a rotating window method as described above in the GO analysis (*p*<0.005). To remove the redundancy in PWMs, we grouped the PWMs into TF families according to their classifications in the TRANSFAC database and we averaged the associated circadian phases of significant TF PWMs in the same TF families using the “mean” function in the R “circular” package. The results are summarized in Table S4. The detailed information about TF enrichment and their associations with any specific circadian phase intervals can be found in our website (http://www.picb.ac.cn/circadian/).

### Knockout or Mutant Mouse Microarray Data

We collected microarray data in different tissues or cell types from knockout or mutant mice, including liver and skeletal muscle in a *Clock* mutant, atrium and ventricle in a cardiomyocyte-specific *Clock* mutant, liver in a liver-specific conditional *Nr1d1* mutant, aorta in *Arntl* and *Npas2* knockout or mutant, liver in a *Rora*/*Rorc* knockout, liver and kidney in a *Dbp*/*Hlf*/*Tef* knockout, liver in a *Ppara*-null mice on Sv129 background treated by the *Ppara* agonist Wy14643, NIH 3T3 cells under *Cebpa/b/d/e* transfection, S49 cells in a *Pka* knockout under cAMP stimulation, cortex and thymus in a *Egr1*/*Egr3* knockout, liver and primary chrodrocytes in a *Nr3c1* (glucocorticoid receptor) knockout treated by the glucocorticoid agonist deamethasone (DEX), and embryonic fibroblast in a *Hsf1* knockout under heat shock (Table S5). We also included the microarray experiment in the SCN of mouse exposed to 30 minute light pulse at 1 hour after the light off period compared to a dark pulse [10]. For the knockout or mutant mice microarray data where time series were available, we applied a two-way ANOVA using genotypes and time series as factors. The *p*-values and fold changes in the genotype comparison were used. For the knockout or mutant mice microarray data where external treatments such as Wy14643, cAMP, DEX, and heat were available, we applied a two-way ANOVA using genotypes and treatments as factors. Here the *p*-values and fold changes in cross-interactions between two factors were used. For *Dbp*/*Hlf*/*Tef* and *Egr1*/*Egr3* knockout or mutant and *Cebpa/b/d/e* transfection experiments, we applied one-way ANOVA using genotypes as factor. For *Rora*/*Rorc*, *Arntl*, and *Npas2* knockout or mutant experiments, we applied the LIMMA program using genotypes as the factor. In the *Rora*/*Rorc* knockout or mutant experiment, *Rora* knockout, *Rorc* knockout, *Rora*/*Rorc* double knockout were treated as the same genotype. In *Pka* knockout or mutant experiment, only the data at 0 hr and 2 hr of cAMP stimulation were used to include the directly affected genes in the cAMP signaling cascade. In *Dbp*/*Hlf*/*Tef* knockout or mutant experiments, the averages of log_2_(*p-*value) and log_2_(fold change) in three experiments: triple knockout vs. wild type in liver, triple knockout vs. triple heterozygotes in liver, and triple knockout vs. wild type in kidney were used as the overall log_2_(*p-*value) and log_2_(fold change). *Ppara* knockout data were obtained from the third and fourth study in [22]. To combine the results in third and fourth studies, we extracted the probe sets with consistent log fold changes of *Ppara* knockout effect of both studies. The maximum of *p-*values of both studies and mean of log fold changes were used. For *Egr1*/*Egr3* knockout in cortex and thymus and *Nr3c1* knockout in liver and primary chrodrocytes, the significantly affected gene lists were simply merged in two tissues or cell types. In all knockout or mutant data, a *p-*value less than 0.01 and a |log_2_(fold change)|>0.5 were used to identify the significantly up- or down-regulated genes in the knockout or mutant.

To reliably identify *Arntl*/*Clock* and *Nr1d1*/*Rora*/*Rorc* controlled genes, we combined the evidences from multiple datasets. *Arntl*/*Clock* controlled genes were identified as those satisfying two out of the five conditions: down-regulated in the *Clock* knockout in liver, down-regulated in the *Clock* knockout in skeletal muscle, down-regulated in the cardiomyocyte-specific *Clock* knockout in atria, down-regulated in the cardiomyocyte-specific *Clock* knockout in ventricle, and down-regulated in the *Arntl* knockout in aorta. As *Nr1d1*, a repressor, was significantly down-regulated in the *Arntl* or *Clock* knockout or mutant, the significant up-regulation in the *Arntl* or *Clock* knockout or mutant was also considered to be the evidence for *Nr1d1* controlled genes. Thus, *Nr1d1*/*Rora*/*Rorc* controlled genes were identified as those satisfying one out of the seven conditions: up-regulated in the *Clock* knockout in liver, up-regulated in the *Clock* knockout in skeletal muscle, up-regulated in the cardiomyocyte-specific *Clock* knockout in atria, up-regulated in the cardiomyocyte-specific *Clock* knockout ventricle, up-regulated in the *Arntl* knockout in aorta, up-regulated in the *Nr1d1* conditional knockout, and down-regulated in the *Rora*/*Rorc* knockout. *Dbp*/*Hlf*/*Tef*, *Ppara*, *Egr1*/*Egr3*, *Pka*, *Nr3c1*, and *Hsf1* controlled genes were identified as those that were significantly down-regulated in a knockout or mutant mouse compared to the wild type mouse. CEBP controlled genes were identified as those that were significantly up-regulated in *Cebpa/b/d/e* transfected cells compared to the control cells.

We identified 380 *Arntl*/*Clock*, 1,166 *Nr1d1*/*Rora*/*Rorc*, 53 *Npas2*, 53 *Dbp*/*Hlf*/*Tef*, 627 *Cebp*, 536 *Ppara*, 710 *Egr1*/*Egr3*, 464 *Pka*, 341 *Nr3c1*, and 425 *Hsf1* controlled genes from the knockout or mutant experiments. To identify the direct target genes of transcription factors in knockout or mutant experiments, we required that the significantly affected genes in a knockout or mutant must have at least one putative binding site of their respective transcription factors in the promoter regions. We identified 320 EBOX, 295 RRE (Rev-erb/Ror element), 47 *Npas2*-regulated element, 43 DBOX, 607 CEBP, 516 PPRE (peroxisome proliferator responsive element), 492 EGRE (Egr element), 455 CRE (cAMP response element), 326 GRE (Glucocorticoid response element), and 122 HSE (Heat shock element) directly controlled genes after combining with the promoter analysis (Table S6).

## Supporting Information

Figure S1 Two functions *y*_+_(*x*) = median(*dij*(*rij*>*x*)) and *y*_−_(*x*) = median(*dij*(*rij*<*x*)) are plotted for −1≤*x*≤1, where *rij* is the correlation coefficient between the tissue gene expression profiles and *dij* is the circadian phase differences of the core circadian gene pairs (*i*,*j*).
(0.22 MB TIF)

Figure S2 Comparison of circadian phases among the overlapping circadian genes between mouse liver and rat liver. The genes with *p*<0.01 from the circular ANOVA test are colored in red. The solid line represents *y* = *x*. The dashed lines represent *y* = *x*±4, respectively.
(0.03 MB PDF)

Figure S3 Circadian phase distributions of circadian oscillating genes controlled by 9 *cis*-regulatory elements. The circadian oscillating genes here have consistent circadian phases across multiple tissues (*p*<1/3 in circular range test). (A) EBOX (ARNTL/CLOCK); (B) RRE (NR1D1/NR1D2/RORA/RORC); (C) DBOX (DBP/TEF/NFIL3); (D) CEBP (CEBPA/B/D/E); (E) CRE (PKA); (F) EGRE (EGR1/EGR3); (G) GRE (NR3C1); (H) HSF (HSF1); (I) PPRE (PPARA).
(0.43 MB TIF)

Table S1 Circadian microarray datasets used in this study.
(0.13 MB DOC)

Table S2 Complete list of circadian oscillating genes in 14 mouse tissues.
(2.92 MB XLS)

Table S3 List of significant biological processes associated with circadian phases in different tissues.
(0.11 MB DOC)

Table S4 List of significant TF families associated with circadian phases in different tissues.
(0.04 MB XLS)

Table S5 Summary of TF knockout or mutant mouse microarray experiments.
(0.10 MB DOC)

Table S6 List of gene regulatory interactions identified from TF knockout or mutant microarray experiments and promoter analysis.
(0.08 MB TXT)
