# Supplementary figures and images for: Analysis of Gene Regulatory Networks in the Mammalian Circadian Rhythm
Source: PLoS Comput Biol. 2008 Oct 10;4(10):e1000193. doi: 10.1371/journal.pcbi.1000193 (PMC2543109; doi:10.1371/journal.pcbi.1000193)

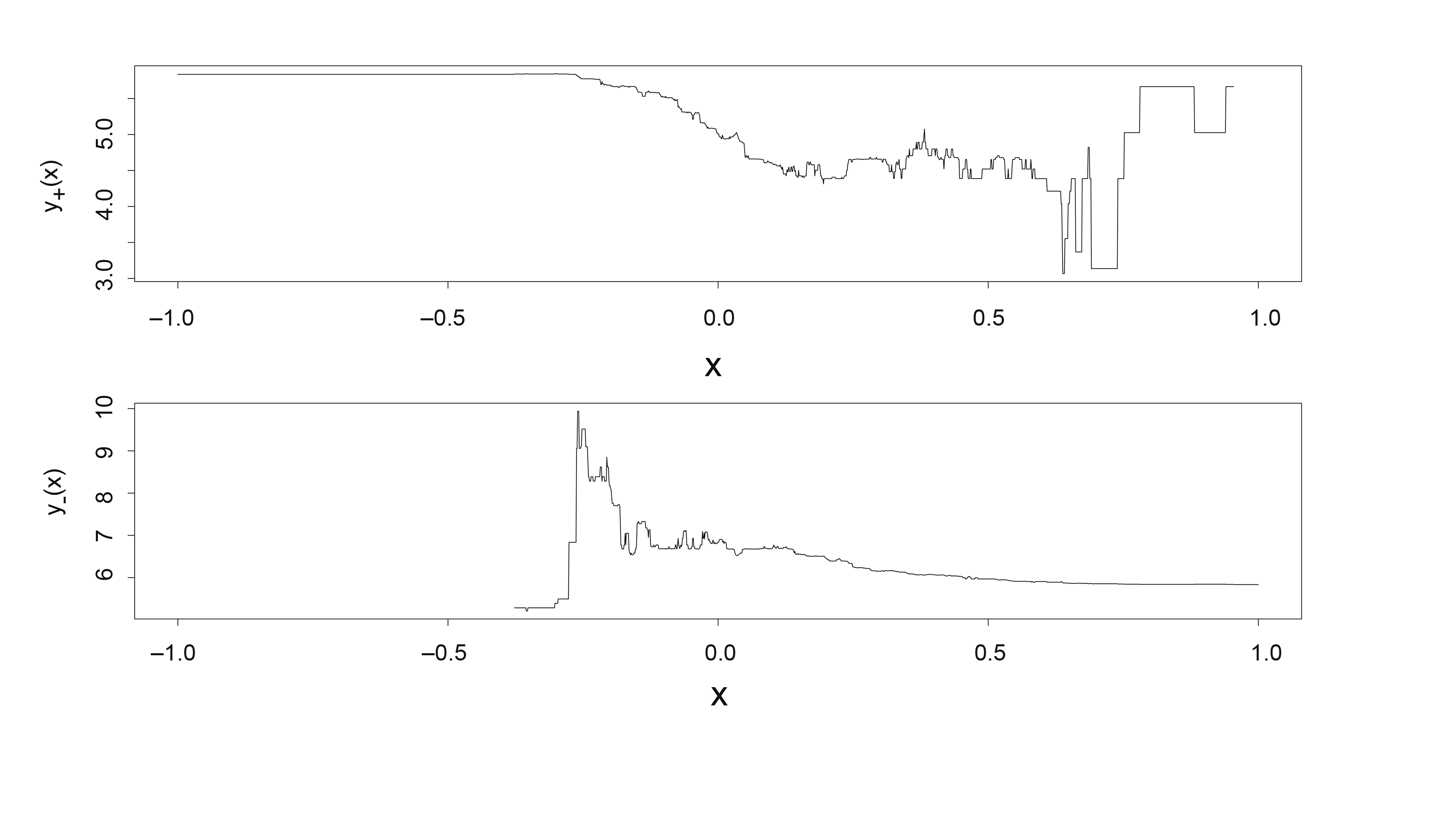

Supplement: Figure S1 — Two functions y +(x) = median(dij(rij>x)) and y −(x) = median(dij(rij<x)) are plotted for −1≤x≤1, where rij is the correlation coefficient between the tissue gene expression profiles and dij is the circadian phase differences of the core circadian gene pairs (i,j). (0.22 MB TIF) [file pcbi.1000193.s001.tif]

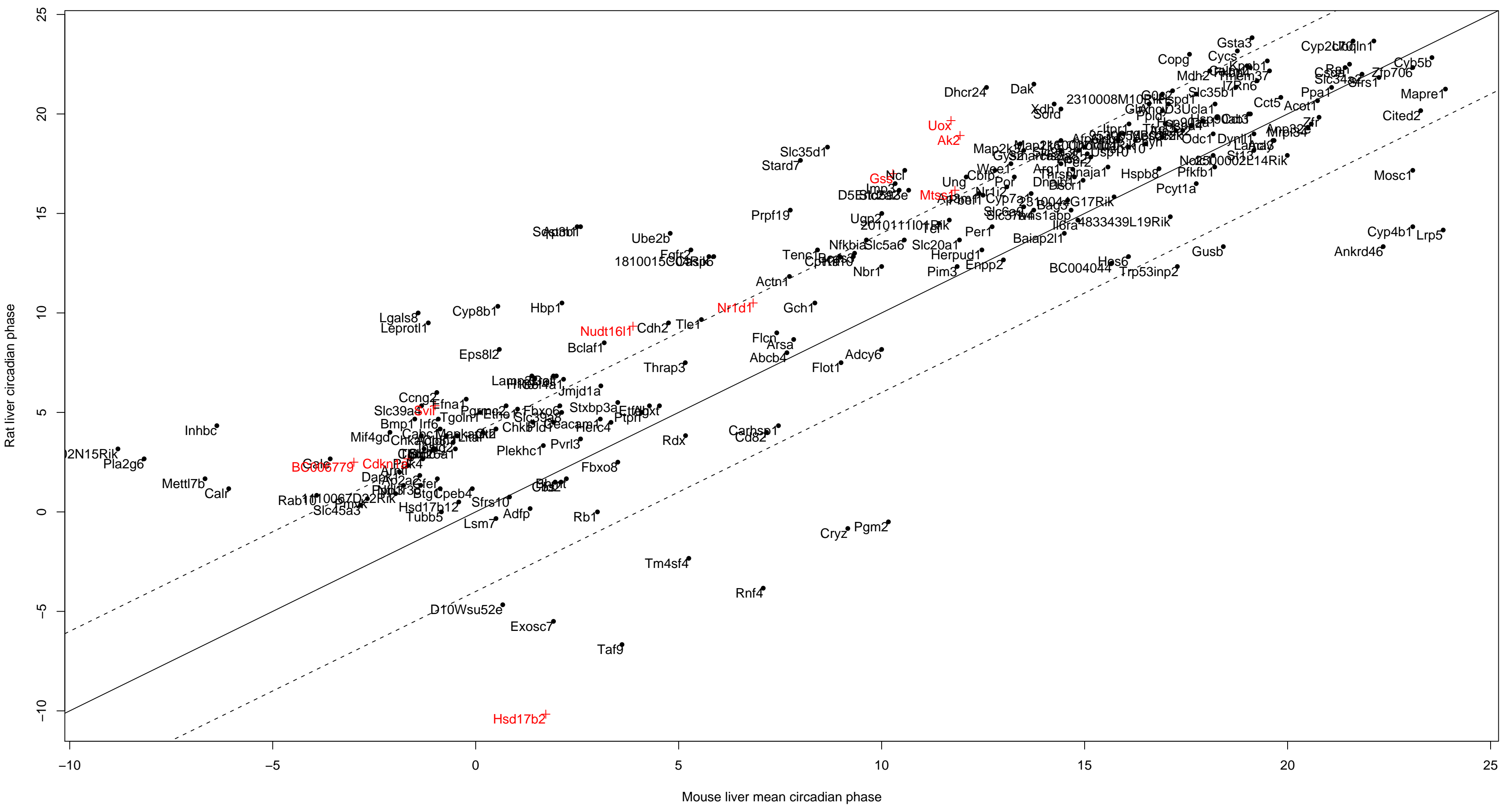

Supplement: Figure S2 — Comparison of circadian phases among the overlapping circadian genes between mouse liver and rat liver. The genes with p<0.01 from the circular ANOVA test are colored in red. The solid line represents y = x. The dashed lines represent y = x±4, respectively. (0.03 MB PDF) [file pcbi.1000193.s002.pdf]

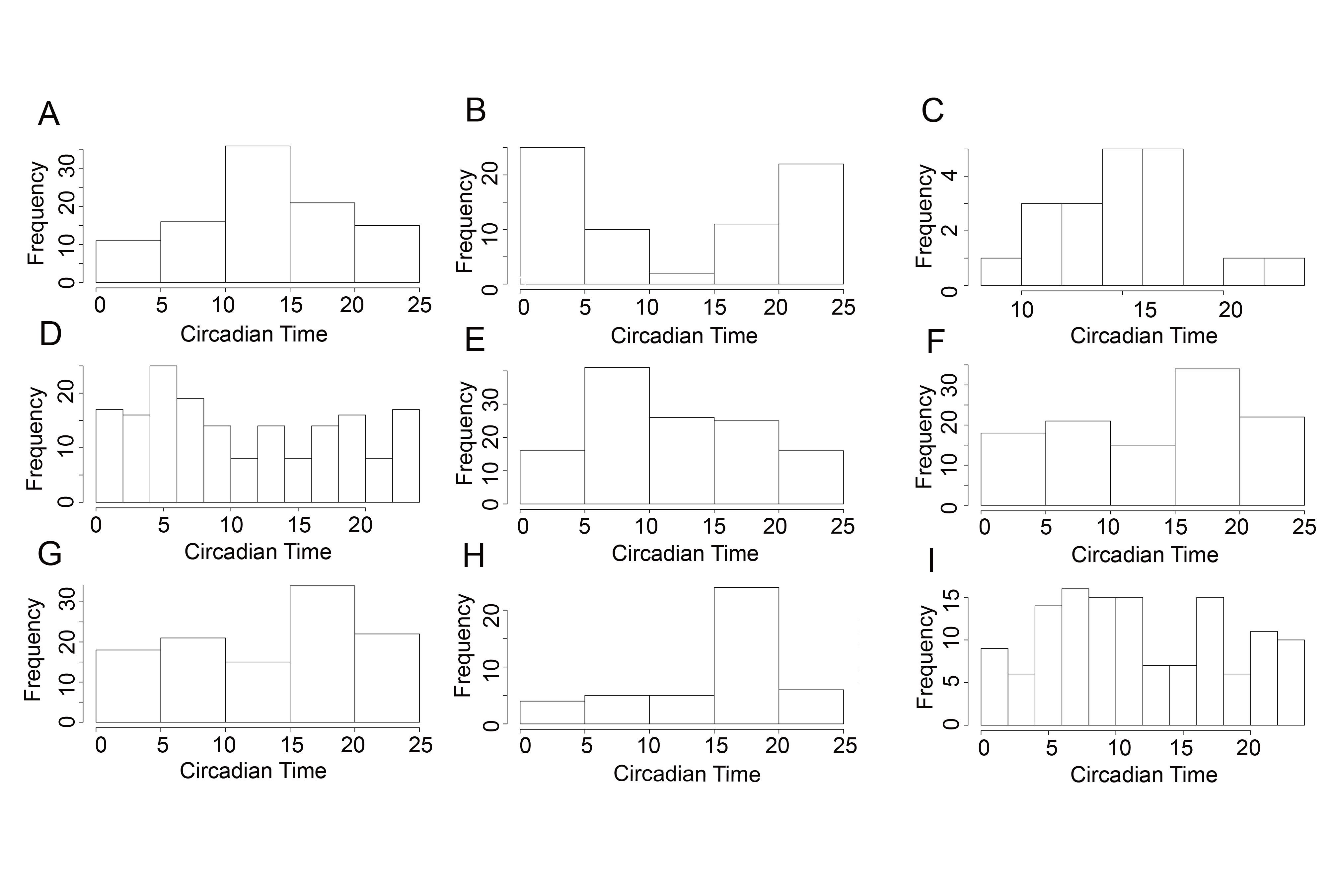

Supplement: Figure S3 — Circadian phase distributions of circadian oscillating genes controlled by 9 cis-regulatory elements. The circadian oscillating genes here have consistent circadian phases across multiple tissues (p<1/3 in circular range test). (A) EBOX (ARNTL/CLOCK); (B) RRE (NR1D1/NR1D2/RORA/RORC); (C) DBOX (DBP/TEF/NFIL3); (D) CEBP (CEBPA/B/D/E); (E) CRE (PKA); (F) EGRE (EGR1/EGR3); (G) GRE (NR3C1); (H) HSF (HSF1); (I) PPRE (PPARA). (0.43 MB TIF) [file pcbi.1000193.s003.tif]
