# Supplementary material for: Analysis of Gene Regulatory Networks in the Mammalian Circadian Rhythm
Source: PLoS Comput Biol. 2008 Oct 10;4(10):e1000193. doi: 10.1371/journal.pcbi.1000193 (PMC2543109; doi:10.1371/journal.pcbi.1000193)
Supplement: Table S1 — Circadian microarray datasets used in this study. (0.13 MB DOC) [file pcbi.1000193.s004.doc]

**Table S1.** Circadian microarray datasets used in this study

| Source | Tissue | Microarray chip type | Data type | Replica at each time point | Time series | Number of circadian oscillating genes | Data source |
| --- | --- | --- | --- | --- | --- | --- | --- |
| Ueda et al. 2002 [1] | Mouse SCN (Balb/c) | mgu74av2 | Gene list and peak time | 1 | Every 4 hours between CT0 and CT44 | 98 | Extracted from literature |
| Panda et al. 2002 [2] | Mouse SCN (C57BL/6J) | mgu74a | Raw cel files | 2 | Every 4 hours between CT18 and CT62 | 1104 | Author provided |
| Ueda et al. 2002 [1] | Mouse Liver | mgu74a | Gene list and peak time | 1 | Every 4 hours between CT0 and CT44 | 387 | Extracted from literature |
| Panda et al. 2002 [2] | Mouse Liver | mgu74a | Raw cel files | 2 | Every 4 hours between CT18 and CT62 | 1039 | Author provided |
| Storch et al. 2002 [3] | Mouse Liver (C57/Bl6) | mgu74av2 | Raw cel files | 1 | Every 4 hours between CT6 and CT50 | 1650 | Author provided |
| Miller et al. 2007 [4] | Mouse Liver (C57BL/6J) | gngnf | Raw cel files | 2 | Every 4 hours between CT18 and CT62 | 1548 | GSE3751 |
| Zvonic et al. 2006 [5] | Mouse Liver (AKR/J) | mouse430a2 | DMTv3.0 normalized files^a^ | 2 | Every 4 hours between CT0 and CT24 | 1531 | Author provided |
| Panda et al. unpublished | Mouse Kidney | Mgu74av2 | MAS4 normalized files | 2 | Every 4 hours between CT18 and CT62 | 658 | Author provided |
| Panda et al. unpublished | Mouse Aorta | Mgu74av2 | MAS4 normalized files | 1 | Every 4 hours between CT18 and CT62 | 1050 | GSE414 |
| Miller et al. 2007 [4] | Mouse Skeletal muscle | GnGnF | Raw cel files | 2 | Every 4 hours between CT18 and CT62 | 440 | GSE3751 |
| Storch et al. 2002 [3] | Mouse Heart | Mgu74av2 | Raw cel files | 1 | Every 4 hours between CT6 and CT50 | 1244 | Author provided |
| Oster et al. 2006 [6] | Mouse adrenal gland (C57BL/6J) | mouse4302 | Dchip1.3 normalized files | 2 | Every 4 hours between CT2 and CT46 | 4162 | GSE4238 |
| Zvonic et al. 2006 [5] | Mouse brown adipose tissue | mouse430a2 | DMTv3.0 normalized files | 2 | Every 4 hours between CT0 and CT24 | 1018 | Author provided |
| Zvonic et al. 2006 [5] | Mouse white adipose tissue | mouse430a2 | DMTv3.0 normalized files* | 2 | Every 4 hours between CT0 and CT24 | 818 | Author provided |
| Zvonic et al. 2007 [7] | Mouse calvarial bone (AKR/J) | mouse430a2 | DMTv3.0 normalized files | 1 | Every 4 hours between CT0 and CT48 | 2352 | Author provided |
| Yang et al. 2007 [8] | Mouse prefrontal Cortex (C57BL/6J) | mouse4302 | Raw cel files | 3 | CT3, CT9, CT15, CT21 | 1209 | GSE9471 |
| Maret et al. 2007 [9] | Mouse whole brain (C57BL/6J, AKR/J, DBA/2J) | Mouse4302 | Raw cel files | 3 | CT6, CT12, CT18, CT0 | 1901 (C56BL/6J),  2098 (AKR/J), 2595 (DBA/2J) | GSE9444 |
| Bray et al. 2007 [10] | Mouse atrium and ventricle | illuminaMousev1 | Illumina normalized data | 4 | Every 4 hours between CT0 and CT21 | 538 (Atrium), 230 (Ventricle) | GSE10045 |
| Almon Richard, unpublished | Rat Liver | Rae230a | Raw cel files | 3 | 18 selected time points between CT0.25 and CT23.75 | 1269 | GSE8988 |
| Almon Richard, unpublished | Rat Skeletal muscle | Rae230a | Raw cel files | 3 | 18 selected time points between CT0.25 and CT23.75 | 803 | GSE8989 |
| Lemos et al. 2006 [11] | Monkey (macaca mulatta) adrenal gland | Hgu133a | Raw cel files | 1 | Every 4 hours between CT0 (7AM) and CT20 (3AM) | 603 | GSE2703 |
| Zambon et al. 2003 [12] | Human Skeletal muscle | Hgu95a | Raw cel files | 4 | CT1 (8AM) vs. CT13 (8PM) | 235 | GSE183 |

^*^DMTv3.0: Data Mining Tool v3.0 (Affymetrix)

1. Ueda HR, Chen W, Adachi A, Wakamatsu H, Hayashi S, et al. (2002) A transcription factor response element for gene expression during circadian night. Nature 418: 534-539.

2. Panda S, Antoch MP, Miller BH, Su AI, Schook AB, et al. (2002) Coordinated transcription of key pathways in the mouse by the circadian clock. Cell 109: 307-320.

3. Storch KF, Lipan O, Leykin I, Viswanathan N, Davis FC, et al. (2002) Extensive and divergent circadian gene expression in liver and heart. Nature 417: 78-83.

4. Miller BH, McDearmon EL, Panda S, Hayes KR, Zhang J, et al. (2007) Circadian and CLOCK-controlled regulation of the mouse transcriptome and cell proliferation. Proc Natl Acad Sci U S A 104: 3342-3347.

5. Zvonic S, Ptitsyn AA, Conrad SA, Scott LK, Floyd ZE, et al. (2006) Characterization of peripheral circadian clocks in adipose tissues. Diabetes 55: 962-970.

6. Oster H, Damerow S, Hut RA, Eichele G (2006) Transcriptional profiling in the adrenal gland reveals circadian regulation of hormone biosynthesis genes and nucleosome assembly Genes. J Biol Rhythms 21: 350-361.

7. Zvonic S, Ptitsyn AA, Kilroy G, Wu X, Conrad SA, et al. (2007) Circadian oscillation of gene expression in murine calvarial bone. J Bone Miner Res 22: 357-365.

8. Yang S, Wang K, Valladares O, Hannenhalli S, Bucan M (2007) Genome-wide expression profiling and bioinformatics analysis of diurnally regulated genes in the mouse prefrontal cortex. Genome Biol 8: R247.

9. Maret S, Dorsaz S, Gurcel L, Pradervand S, Petit B, et al. (2007) Homer1a is a core brain molecular correlate of sleep loss. Proc Natl Acad Sci U S A 104: 20090-20095.

10. Bray MS, Shaw CA, Moore MWS, Garcia RAP, Zanquetta MM, et al. (2007) Disruption of the circadian clock within the cardiomyocyte influences myocardial contractile function, metabolism, and gene expression. Am J Physiol Heart Circ Physiol: 01291.02007.

11. Lemos DR, Downs JL, Urbanski HF (2006) Twenty-four-hour rhythmic gene expression in the rhesus macaque adrenal gland. Mol Endocrinol 20: 1164-1176.

12. Zambon AC, McDearmon EL, Salomonis N, Vranizan KM, Johansen KL, et al. (2003) Time- and exercise-dependent gene regulation in human skeletal muscle. Genome Biol 4: R61.
