# Supplementary material for: Analysis of Gene Regulatory Networks in the Mammalian Circadian Rhythm
Source: PLoS Comput Biol. 2008 Oct 10;4(10):e1000193. doi: 10.1371/journal.pcbi.1000193 (PMC2543109; doi:10.1371/journal.pcbi.1000193)
Supplement: Table S3 — List of significant biological processes associated with circadian phases in different tissues. (0.11 MB DOC) [file pcbi.1000193.s006.doc]

**Table S3.** List of significant biological processes associated with circadian time in different tissues

| Tissue | Biological Process | Associated Circadian Time |
| --- | --- | --- |
| SCN | ATP synthesis | 19.9 |
| SCN | protein translation | 20.4 |
| SCN | transport | 10.2 |
| SCN | heat shock response | 15.3 |
| Liver | coagulation | 4 |
| Liver | carbohydrate metabolism | 17 |
| Liver | amino acid metabolism | 15.4 |
| Liver | steroid biosynthesis | 1.3 |
| Liver | heat shock response | 15.9 |
| Kidney | electron transport | 13.3 |
| Kidney | transport | 5.86 |
| Kidney | steroid biosynthesis | 23 |
| Kidney | heat shock response | 16.3 |
| Aorta | immune response | 0.5 |
| Aorta | lipid metabolism | 7.9 |
| Aorta | vitamin metabolism | 15.2 |
| Aorta | protein folding | 19.8 |
| Aorta | transport | 7.5 |
| Skeletal muscle | transcription regulation | 11.5 |
| Skeletal muscle | phosphate transport | 1 |
| Heart | cell proliferation | 22.9 |
| Heart | muscle contraction | 9.5 |
| Heart | organ development | 1.2 |
| Adrenal | steroid biosynthesis | 17.9 |
| Adrenal | lipid metabolism | 17.9 |
| Adrenal | heme metabolism | 13.8 |
| Adrenal | RNA splicing | 21.1 |
| Adrenal | heat shock response | 18.3 |
| Adrenal | transcription regulation | 7.7 |
| Adrenal | Ras signaling | 9.3 |
| Adrenal | organ development | 9.89 |
| Brown adipose tissue | steroid biosynthesis | 22 |
| Brown adipose tissue | lipid metabolism | 22.2 |
| Brown adipose tissue | protein folding | 15.8 |
| Brown adipose tissue | organ development | 21.8 |
| White adipose tissue | steroid biosynthesis | 22.2 |
| White adipose tissue | lipid metabolism | 22 |
| White adipose tissue | protein translation | 8.9 |
| White adipose tissue | protein folding | 16 |
| Calvarial bone | hair cycle | 21.7 |
| Calvarial bone | synaptic transmission | 2.5 |
| Calvarial bone | neurogenesis | 2.5 |
| Calvarial bone | regulation of apoptosis | 23.5 |
| Calvarial bone | protein folding | 13.4 |
| Prefrontal cortex | negative regulation of protein kinase | 16.6 |
| Prefrontal cortex | development | 2.7 |
| Prefrontal cortex | protein metabolism | 9.5 |
| Whole brain | negative regulation of protein kinase | 21.1 |
| Whole brain | RNA splicing | 16.7 |
| Whole brain | transcription regulation | 16.9 |
| Whole brain | protein folding | 20.4 |
