# Supplementary material for: Analysis of Gene Regulatory Networks in the Mammalian Circadian Rhythm
Source: PLoS Comput Biol. 2008 Oct 10;4(10):e1000193. doi: 10.1371/journal.pcbi.1000193 (PMC2543109; doi:10.1371/journal.pcbi.1000193)
Supplement: Table S5 — Summary of TF knockout or mutant mouse microarray experiments. (0.10 MB DOC) [file pcbi.1000193.s008.doc]

**Table S5.** Summary of TF knockout or mutant microarray experiments.

| TF name | Tissue/Cell | Treatment | Reference | Method | Array format and data source |
| --- | --- | --- | --- | --- | --- |
| Clock | Liver/SKM | C57BL/6J *Clock* homozygous mutant mice at CT22-CT46 | [1] | Two-way ANOVA p<0.01 in CLOCK vs. WT comparison | Gngnf (GSE3746 & GSE3748) |
| Clock | Atrium/Ventricle | cardiomyocyte-specific circadian clock mutant (CCM) | [2] | Two-way ANOVA p<0.01 in CLOCK vs. WT comparison | illuminaMousev1  (GSE10045) |
| Nr1d1 | Liver | LAP-tTA/TRE-Rev-erbα  Double transgenic mice | [3] | Two-way ANOVA p<0.01 in transgenic vs. WT comparison | Mouse4302 (E-MEXP-842) |
| Rora/Rorc | Liver | *RORα^sg/sg^, RORγ*^-/-^, DKO mice | [4] | Limma p<0.01 | Aglient (GSE7564) |
| Dbp/Hlf/Tef | Liver/Kidney | *Dbp^-/-^Hlf^-/-^Tef^-/-^, Dbp^+/-^Hlf^+/-^Tef^+/-^* | [5] | ANOVA combined p<0.01 | Mouse4302 (E-MEXP-565) |
| Arntl/Npas2 | Aorta | *Bmal1*^-/-^*, Npas2^mut^* | [6] | Limma p<0.01 | Mouse430a2 (GSE3849 & GSE3850) |
| Ppara | Liver | PPARα-null mice on Sv129 background treated by Wy14643 | [7] | Two-way ANOVA p<0.01 (cross-interaction). Combination of study 3 and 5. | Mouse4302 (GSE8292 & GSE8302) |
| Egr1/3 | Cortex/Thymus | EGR1/3 double knockout | [8] | ANOVA p<0.01 3 vs. 3 comparison | Mouse4302 (GSE5587 & GSE4752) |
| PKA | S49 cells | Kin^-^ S49 cell | [9] | ANOVA p<0.01 (cross-interaction) at 2 hours after cAMP simulation | Mouse430a2 (GSE2413) |
| Cebpa/b/d/e | NIH 3T3 cells | CEBP transfection | [10] | ANOVA p<0.01 | Mgu74av2 (GSE2188) |
| Nr3c1 | Liver | SCN ablated mice treated by dexamethasone at CT11, 23, 35. | [11] | Dexamethasone treated vs. vehicle treated ANOVA p<0.01 (cross-interaction) | Mgu74av2 (GSE564) |
| Nr3c1 | Primary chrodrocytes | Dexamethasone treated for 6 and 24 hours | [12] | Dexamethasone treated vs wildtype ANOVA p<0.01 (cross-interaction) at 6hrs and 24hrs | Mouse430a2 (GSE7683) |
| Hsf1 | Embryonic Fibroblast | HSF1^-/-^ heat shock for 0, 0.5, 1, 2, 3, 4, 6, 8hrs | [13] | ANOVA p<0.01 (cross-interaction) | cDNA array (GSE3074) |

1. Miller BH, McDearmon EL, Panda S, Hayes KR, Zhang J, et al. (2007) Circadian and CLOCK-controlled regulation of the mouse transcriptome and cell proliferation. Proc Natl Acad Sci U S A 104: 3342-3347.

2. Bray MS, Shaw CA, Moore MWS, Garcia RAP, Zanquetta MM, et al. (2007) Disruption of the circadian clock within the cardiomyocyte influences myocardial contractile function, metabolism, and gene expression. Am J Physiol Heart Circ Physiol: 01291.02007.

3. Kornmann B, Schaad O, Bujard H, Takahashi JS, Schibler U (2007) System-driven and oscillator-dependent circadian transcription in mice with a conditionally cctive liver clock. PLoS Biology 5: e34.

4. Kang HS, Angers M, Beak JY, Wu X, Gimble JM, et al. (2007) Gene expression profiling reveals a regulatory role for ROR{alpha} and ROR{gamma} in phase I and phase II metabolism. Physiol Genomics 31: 281-294.

5. Gachon F, Olela FF, Schaad O, Descombes P, Schibler U (2006) The circadian PAR-domain basic leucine zipper transcription factors DBP, TEF, and HLF modulate basal and inducible xenobiotic detoxification. Cell Metabolism 4: 25-36.

6. Curtis AM, Cheng Y, Kapoor S, Reilly D, Price TS, et al. (2007) Circadian variation of blood pressure and the vascular response to asynchronous stress. Proc Natl Acad Sci U S A 104: 3450-3455.

7. Maryam Rakhshandehroo LS, MerjaMatilainen, Rinke Stienstra,, Carsten Carlberg PJdG, Michael Muller, and Sander Kersten (2007) Comprehensive analysis of PPARα-dependent regulation of hepatic lipid metabolism by expression profiling. PPAR Research 2007: Article ID 26839, 26813 pages.

8. Carter JH, Lefebvre JM, Wiest DL, Tourtellotte WG (2007) Redundant role for early growth response transcriptional regulators in thymocyte differentiation and survival. J Immunol 178: 6796-6805.

9. Zambon AC, Zhang L, Minovitsky S, Kanter JR, Prabhakar S, et al. (2005) Gene expression patterns define key transcriptional events in cell-cycle regulation by cAMP and protein kinase A. Proc Natl Acad Sci U S A 102: 8561-8566.

10. Gery S, Gombart AF, Yi WS, Koeffler C, Hofmann W-K, et al. (2005) Transcription profiling of C/EBP targets identifies Per2 as a gene implicated in myeloid leukemia. Blood 106: 2827-2836.

11. Reddy AB, Maywood ES, Karp NA, King VM, Inoue Y, et al. (2007) Glucocorticoid signaling synchronizes the liver circadian transcriptome. Hepatology 45: 1478-1488.

12. James C, Ulici V, Tuckermann J, Underhill TM, Beier F (2007) Expression profiling of Dexamethasone-treated primary chondrocytes identifies targets of glucocorticoid signalling in endochondral bone development. BMC Genomics 8: 205.

13. Trinklein ND, Murray JI, Hartman SJ, Botstein D, Myers RM (2004) The role of heat shock transcription factor 1 in the genome-wide regulation of the mammalian heat shock response. Mol Biol Cell 15: 1254-1261.
